# Frequency-Dependent Neural Activity in Patients with Unilateral Vascular Pulsatile Tinnitus

**DOI:** 10.1155/2016/4918186

**Published:** 2016-06-20

**Authors:** Han Lv, Pengfei Zhao, Zhaohui Liu, Guopeng Wang, Rong Zeng, Fei Yan, Cheng Dong, Ling Zhang, Rui Li, Peng Wang, Ting Li, Shusheng Gong, Zhenchang Wang

**Affiliations:** ^1^Department of Radiology, Beijing Friendship Hospital, Capital Medical University, Beijing 100050, China; ^2^Neuroradiology Division, Department of Radiology, Stanford University, Stanford, CA 94305, USA; ^3^Department of Radiology, Beijing Tongren Hospital, Capital Medical University, Beijing 100730, China; ^4^Department of Otolaryngology Head and Neck Surgery, Beijing Friendship Hospital, Capital Medical University, Beijing 100050, China

## Abstract

Previous resting-state functional magnetic resonance imaging (rs-fMRI) studies have shown that neurological changes are important findings in vascular pulsatile tinnitus (PT) patients. Here, we utilized rs-fMRI to measure the amplitude of low-frequency fluctuations (ALFF) in forty patients with unilateral PT and forty age-, gender-, and education-matched normal control subjects. Two different frequency bands (slow-4, 0.027–0.073 Hz, and slow-5, 0.010–0.027 Hz, which are more sensitive to subcortical and cortical neurological signal changes, resp.) were analyzed to examine the intrinsic brain activity in detail. Compared to controls, PT patients had increased ALFF values mainly in the PCu, bilateral IPL (inferior parietal lobule), left IFG (inferior frontal gyrus), and right IFG/anterior insula and decreased ALFF values in the multiple occipital areas including bilateral middle-inferior occipital lobe. For the differences of the two frequency bands, widespread ALFF differences were observed. The ALFF abnormalities in aMPFC/ACC, PCu, right IPL, and some regions of occipital and parietal cortices were greater in the slow-5 band compared to the slow-4 band. Additionally, the THI score of PT patients was positively correlated with changes in slow-5 and slow-4 band in PCu. Pulsatile tinnitus is a disease affecting the neurological activities of multiple brain regions. Slow-5 band is more sensitive in detecting the alternations. Our results also indicated the importance of pathophysiological investigations in patients with pulsatile tinnitus in the future.

## 1. Introduction

Tinnitus is defined as the perception of sound without external stimuli. It affects millions of people in the world. About 5%~15% of the world's population has tinnitus [1–6]. Tinnitus can be divided into pulsatile tinnitus (PT) and nonpulsatile tinnitus (NPT). PT coincides with the patient's heartbeat, and the characterized cardiac-synchronous sound described by PT patients can be suppressed by compressing the internal carotid artery or internal jugular vein on the symptomatic side [7, 8], whereas NPT is continuous ringing sound. Among the tinnitus patients, 4% of them are experiencing a pulsatile form of tinnitus [9]. Thus, it is estimated that there are almost twenty million PT patients all over the world.

According to previous studies, patients with PT have both structural and functional abnormalities compared with healthy controls. The structural changes are usually considered as the etiology of PT, such as focal bone defect in the region of the sigmoid sinus, persistent petrosquamosal sinus, sigmoid sinus diverticulum, mastoid emissary vein, atherosclerosis, and dural arteriovenous fistula (dAVF); paraganglioma (e.g., glomus jugulare tumors) and involuntary contraction of muscles in the middle ear are also considered as causes of PT [9–16]. Abnormal blood flow induced by a focal bone defect in the region of the sigmoid sinus is a common etiology [9–16]. The relatively clear etiologies set a valid foundation for us to study the neural activity of PT patients.

Thus, all of the functional activity changes of the brain were considered as results following the tinnitus sound stimulation in PT patients. Following numerous previous neuroimaging studies, resting-state functional magnetic resonance imaging (rs-fMRI) has been proven to be a useful tool for characterizing the intrinsic brain activity in patients with tinnitus [17–22]. This method also provided an efficient and noninvasive way to research the neuropsychological changes of PT [23–25]. For the patients whose etiologies were confirmed as a focal defect of mastoid bone shell in the region of the transverse-sigmoid junction, the increased amplitude of low-frequency fluctuation (ALFF) in precuneus could reflect tinnitus-related distress [23]. The bilaterally increased ALFF value in IFG (inferior frontal gyrus) was considered to be related to tinnitus awareness [24]. These results remind us that, apart from the structural investigations, functional investigations should also be valued in PT patients. However, our last researches were only starts. More methods should be applied to study the neural activity of PT patients in different aspects.

To study the ALFF in detail, Buzsáki and Draguhn [26] divided the low-frequency range into four distinct bands. Zuo et al. reported that slow-4 (0.027–0.073 Hz) and slow-5 (0.010–0.027 Hz) bands reflect mainly white matter and gray matter signals, respectively, while slow-2 (0.198–0.25 Hz) and slow-3 (0.073–0.198 Hz) mainly reflect white matter signals and high-frequency physiological noises [27]. A spectrum-specific analysis of healthy subjects provided a new analytical strategy for the study of the brain [28]. Several researchers have reported healthy subjects [27, 28], patients with amnestic mild cognitive impairment [29], Alzheimer's disease [30], Parkinson's disease [31], and schizophrenia [32] demonstrated widespread frequency band dependent abnormalities in the brains. It is still unknown whether altered ALFF in patients with PT are associated with specific frequency bands and which frequency band is more sensitive in detecting PT-related neural changes in the brain.

In the current study, we applied rs-fMRI to study the changes of ALFF in PT patients within two specific frequency ranges (slow-4, 0.027–0.073 Hz; slow-5, 0.010–0.027 Hz). Our purpose was to examine whether PT-related neural changes are associated with specific frequency bands and which frequency band is more sensitive in exploring PT-related neural changes. We hypothesized that PT patients show abnormal ALFF of intrinsic brain activity in PCC/precuneus, IFG, and so forth, which may partially corresponded with our recent studies [23, 24].

## 2. Subjects and Methods

### 2.1. Subjects

Forty patients with right-sided unilateral PT and forty healthy controls were enrolled in this study. They were matched in age, gender, education years, and right or left handedness. They described the PT sounds as low-pitched, cardiac-synchronous sounds like the beat of a drum, and the sounds can be suppressed by compression of the right neck in the area of internal jugular vein. CTA/V (CT Arteriography and Venography) as well as DSA (digital subtraction angiography) examinations confirmed the etiology of focal bone defect in the region of the sigmoid sinus. Other possible etiologies could be excluded by these examinations [8, 9, 33, 34]. The medical history, symptom, and clinical examinations (puretone audiometry examination) were carefully reviewed to excluded NPT and hearing loss patients. All of the patients and healthy controls did not present with persistent NPT or any degree of hearing loss. (For the definition of the normal level of hearing thresholds: subjects had hearing thresholds <25 dB HL at 0.25, 0.5, 1, 2, 3, 4, 6, and 8 kHz frequencies in the puretone audiometry examination.) All patients were asked to fill Tinnitus Handicap Inventory (THI) [35, 36] to assess tinnitus-related distress and the severity of tinnitus. Higher score refers to a higher degree of tinnitus-related distress. This study was approved by the Research Ethics Committee of Beijing Friendship Hospital and Beijing Tongren Hospital, Capital Medical University. All subjects gave their written informed consent for this study.

### 2.2. MRI Scanning

All of the anatomical and rs-fMRI images were acquired with a General Electric (GE) 3.0 Tesla Trio scanner (Milwaukee, WI, USA). During the scan, subjects were asked to remain motionless in the scanner with eyes closed. Structural images were collected by 3D T1 scans for each subject. The sequence was acquired with the following parameters: slices = 196; slice thickness = 1.0 mm (without gap); field of view = 240 × 240 mm; matrix = 256 × 256; TR/TE/TI (repetition time/echo time/inversion time) = 8.8/3.5/450 ms; and flip angle = 15°. Functional images were also obtained using EPI (echo planar imaging) sequence with the following parameters: number of slices = 28; repetition time = 2,000 ms; echo time = 35 ms; flip angle = 90°; slice thickness = 4 mm; matrix = 64 × 64; and field of view = 24 cm × 24 cm. Each rs-fMRI session lasted 400 seconds.

### 2.3. Data Preprocessing and ALFF Analyses

Functional data preprocessing was performed with DPARSF (Data Processing Assistant for rs-fMRI; http://www.restfmri.net/) [37] and REST (rs-fMRI data analysis toolkit; http://www.restfmri.net/) [38]. Each fMRI series contained 200 time points. The first twenty time points were removed for signal equilibrium and participants' adaptation to the scanning noise. Any subjects with a head motion more than 1.5 mm translation or 1.5 degrees of rotation were excluded. After slice-time correction, the images were further spatially normalized to the MNI (Montreal Neurological Institute) space by applying the transformation parameters obtained from the structural images and resampled into 3 mm isotropic voxel, smoothed with a 3 mm FWHM (full-width at half maximum) Gaussian kernel. Then, linear drift was removed. After data preprocessing, the ALFF was calculated with the same procedure reported previously [39–41]. The time-domain data was first transformed to a frequency domain using a FFT (Fast Fourier Transform). The square root of the power spectrum was computed at each frequency and then averaged across a predefined frequency interval. ALFF was defined as this averaged square root at the given voxel, and results were further divided by the global mean ALFF value in order to reduce the global effects of variability across the subjects [40]. All of the ALFF analyses were based on the GM mask generated above. In our study, we only computed ALFF in the slow-4 (0.027–0.073 Hz) and slow-5 (0.010–0.027 Hz) bands.

As the regional ALFF results could be influenced by gray matter (GM) volume [42, 43], the results of structural images will be used as covariates for ALFF calculations in the following statistical analysis. Also all of the calculations were within the GM mask to exclude the effects from white matter and cerebrospinal fluid.

### 2.4. Statistical Analysis

Demographic data were compared by two-sample *t*-tests and Fisher's exact test using SPSS 12.0 software (SPSS, Inc., Chicago, IL). *P* values < 0.05 were considered to be statistically significant.

To determine the effects of group and frequency band on ALFF, a two-way repeated-measure ANOVA was performed with SPM8 software. We also investigated the relationship between ALFF values of the brain areas showing significant differences and clinical data of the patients. Firstly, we saved each brain area showing significant differences as a mask. Then, we extracted the signal intensity in the PT patients group within each mask we created. After that we could compute Pearson's correlation coefficients between the signal intensity and clinical data of the PT patients using SPSS 12.0 software. *P* values < 0.05 and a cluster size of 27 voxels (corrected for multiple comparisons using Monte Carlo simulation (single voxel *P* = 0.05, simulations = 5000, cluster connection radius *r* = 5 mm, and FWHM = 4 mm, with a resolution of 3 mm × 3 mm × 3 mm)) were considered to be statistically significant. For the clusters showing significant main effects and an interaction between group and frequency band, we performed the* post hoc* two-sample *t*-tests. Results were shown by the REST Slice Viewer (http://www.restfmri.net/).

## 3. Results

### 3.1. Characteristics of the Participants

Eighty participants (forty PT patients and forty healthy control subjects) were recruited for this study. Both groups are statistically comparable for age, gender, education, handedness, and the degree of hearing loss. The characteristics of the subjects are presented in Table 1.

### 3.2. ALFF Analysis

**Figure 1:**
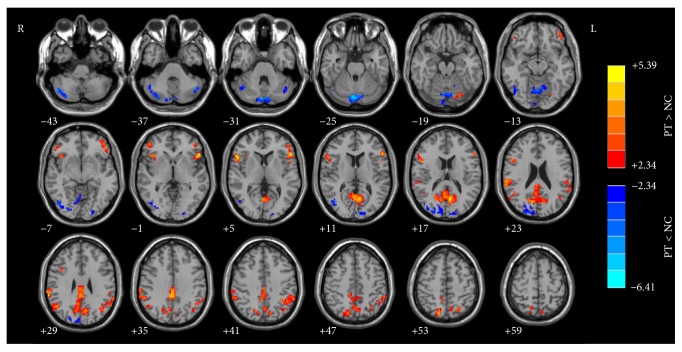
The main effect for group on ALFF. The hot color represents a higher ALFF in PT patients than in the healthy controls. ALFF, amplitude of low-frequency fluctuation; PT, pulsatile tinnitus.

**Figure 2:**
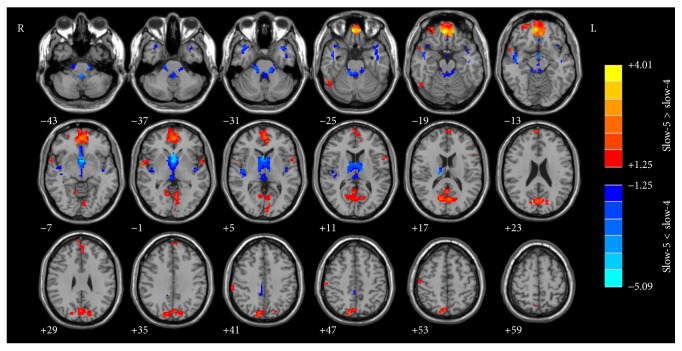
The main effect for frequency band on ALFF. The hot color represents a higher ALFF in the slow-5 band than in the slow-4 band, whereas the cool color represents a lower ALFF. ALFF, amplitude of low-frequency fluctuation.

The results of structural images were used as covariates for ALFF calculations. Figures 1 and 2 show the main effects for group and for frequency band from the two-way repeated-measures ANOVA.

For the brain regions with a main effect of group (Figure 1), the PT patients showed significantly increased ALFF values in PCu, bilateral IPL (inferior parietal lobule), left IFG (inferior frontal gyrus), right IFG/anterior insula, bilateral superior temporal gyrus, and left fusiform gyrus and decreased ALFF values mainly in the multiple occipital areas including bilateral middle-inferior occipital lobe, cuneus, vermis, and part of bilateral cerebellum posterior lobe.

Figure 2 shows the brain regions of the ALFF differences between the frequency bands (slow-5 versus slow-4), including the aMPFC (anterior medial prefrontal cortex)/ACC (anterior cingulate cortex), PCu (precuneus), part of the lateral regions of bilateral superior temporal gyrus, right fusiform gyrus, right postcentral gyrus (slow-5 band > slow-4 band), and basal ganglia and bilateral superior temporal gyrus (slow-5 band < slow-4 band). We noticed that the brain regions with higher ALFF values in the slow-5 band compared with slow-4 band are partially overlapped with the DMN (default-mode networks).

**Figure 3:**
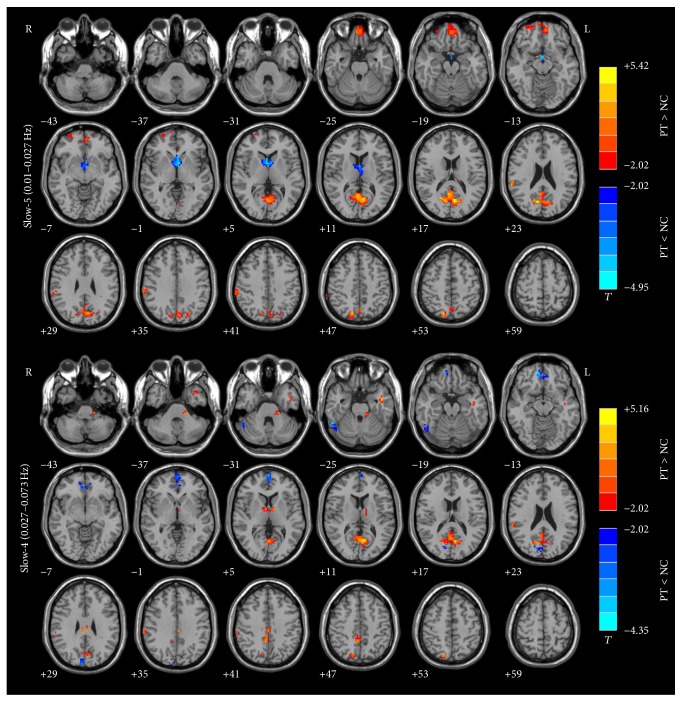
The interaction between frequency band and group on ALFF (*post hoc*). Group differences in the aMPFC/ACC, PCu, right IPL, and some regions of occipital and parietal cortices and cerebellum showed group differences mainly in slow-5 band results. ALFF, amplitude of low-frequency fluctuation; MPFC, medial prefrontal cortex; ACC, anterior cingulate cortex; PCu, precuneus; IPL, inferior parietal lobule.

ALFF abnormalities in the aMPFC/ACC, PCu, right IPL, and some regions of occipital and parietal cortices and cerebellum exhibited group differences in slow-5 band compared to the slow-4 band (Figure 3). The results were obtained by a two-way repeated-measure ANOVA and a* post hoc* test.

**Figure 4:**
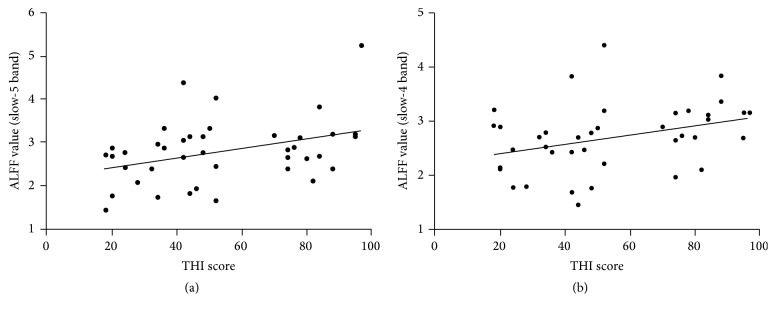
Correlation between the THI score and ALFF value in the PCu of PT patients. (a) Correlation maps in the slow-5 band. (b) Correlation maps in the slow-4 band. THI, Tinnitus Handicap Inventory; ALFF, amplitude of low-frequency fluctuation; PCu, precuneus; PT, pulsatile tinnitus.

We found that PCu with ALFF changes in slow-4 (*r* = 0.342, *P* = 0.031) and slow-5 (*r* = 0.368, *P* = 0.019) bands had significant correlations with the clinical data of PT patients as measured using THI scores (Figures 4(a) and 4(b), resp.).

## 4. Discussion

To our knowledge, this study employed rs-fMRI to investigate spontaneous baseline brain activity changes in patients with pulsatile tinnitus in two different frequency bands (slow-4 and slow-5) for the first time. We found that many brain regions showed significant differences in ALFF at each frequency band and in two subject groups (PT and healthy controls). Additionally, there were several brain regions (aMPFC/ACC, PCu, right IPL, and some regions of occipital and parietal cortices) that exhibited interaction between frequency band and group, where the group differences in the slow-5 band were more significant than those in the slow-4 band. Finally, we found that the precuneus with ALFF changes had significant correlations with the clinical data of PT patients as measured using THI scores. Our study suggests that the ALFF abnormalities of intrinsic brain activity in PT patients are associated with specific frequency bands and may have clinical relevance.

In accordance with previous examinations [27–31], our project presented the distinction between the two frequency bands (slow-4 and slow-5). However, our opinion is the same as Xue et al. [28]: “whether 0.027 Hz is the best breaking point for frequency division should be carefully determined.” The definitions of frequency bands and subbands are still a matter of debate in the extant EEG literatures. Xue et al. discussed this issue in detail [28]. Considering that this cutoff frequency had been applied in previous studies [27–31], we still employed slow-4 and slow-5 as subbands in the brain research of PT patients this time. Future rs-fMRI research should address the spectrum-specific analytical strategy in much more detail.

### 4.1. Differences in ALFF between Groups

We showed that the PCu had increased ALFF in PT patients (Figure 1). PCu is a brain region outside the auditory-perceptual system, playing a central role in tinnitus-related distress [19, 20, 44, 45]. Actually, up to 60% of tinnitus patients have been reported to suffer from different degrees of depression [20, 21, 46–49]. Previous EEG studies of nonpulsatile tinnitus patients showed that the PCu was active within the alpha frequency band in patients with tinnitus-related distress [50]. Highly distressed tinnitus patients demonstrated higher activation in PCu after perception of tinnitus-related sentences (expected to be a kind of stimulus material to activate the brain areas related to tinnitus annoyance) [20]. We also found a positive correlation between THI score and ALFF alternation in slow-4 and slow-5 (Figures 4(a) and 4(b), resp.), providing further support for the tinnitus-related distress analysis. Thus, activated ALFF in PCu represent tinnitus-related distress in PT patients. But why there were different relationships between the THI scores and ALFF at different frequency bands requires additional investigation.

Left IFG and right IFG/anterior insula were also activated in patients with PT. The role of IFG in patients with tinnitus remains unclear. There was a task-fMRI study on NPT patients [51] that showed that, after stimulation at the tinnitus frequency, significantly increased signal intensity was found in the bilateral IFG. The BOLD signal change showed positive correlation with the tinnitus loudness ratings and tinnitus awareness ratings. The IFG was critical for response inhibition. Its activation is closely related to tinnitus awareness [52]. The increased bilateral IFG ALFF values of the PT patients may reflect the inhibitory effort of them to suppress the tinnitus sound.

The insula is divided into three parts, including posterior insula (PI), dorsal anterior insula (dAI), and the ventral anterior insula with distinct connectivity patterns [53]. It is reported to have roles in self-awareness, perception, motor control, interpersonal experience, and cognitive functioning. Different parts of the insula are involved in diverse functions. The anterior insulae (frontoinsular) are functionally connected to ACC, playing a role in emotional control. As part of the limbic system, the frontoinsular part of the brain is considered to play a role in processing distressing information [54, 55]. Increased activation occurs when subjects attempt to suppress their emotions [56]. The increased ALFF in PT patients in the anterior insula might therefore also play a role in suppressing the tinnitus-related distress. Also the frontoinsular part of the brain provides attention switching between tinnitus and other conditions [57, 58]. The increased ALFF values in the frontoinsular part of the brain might reflect its effort to maintain attention to nonauditory events.

The roles of cerebellar posterior lobe and vermis in PT patients are also important. The functional connectivity between the cochlear nuclei and cerebellum indicated the cerebellum association with sound processing [59–61]. For the vermis, it may help us move heads towards the sound source [62, 63]. The decreased ALFF in the vermis may present a downregulation adjustment of its function to avoid misinterpreting the sounds around. Further functional connectivity studies on PT patients are needed to support this hypothesis.

### 4.2. Frequency-Dependent Changes in ALFF in PT Patients and Healthy Controls

Different oscillatory patterns may represent different neurological functions. Analyzing BOLD signals at various spectra could provide more information than ever. There were significant differences between slow-5 and slow-4 bands (Figure 2). In this study, areas of ALFF differences between slow-5 and slow-4 bands included the aMPFC/ACC, PCu, part of the lateral regions of bilateral superior temporal gyrus, right fusiform gyrus, right postcentral gyrus (slow-5 band > slow-4 band), and basal ganglia and bilateral superior temporal gyrus (slow-5 band < slow-4 band). Part of our results was highly consistent with that of previous studies [27–29, 31] (including healthy subjects investigations) on bilateral aMPFC/ACC, PCu, part of the lateral regions of the temporal lobe, and basal ganglia. The differences between these two frequency bands were also present in healthy subjects. Zuo et al. [27] showed that the fALFF (Fractional Amplitude of Low-Frequency Fluctuations) is a kind of local features similar to ALFF. It is defined as the total power within the low-frequency range divided by the total power in the entire detectable frequency range (i.e., 0–0.25 Hz); other calculation procedures are similar (as ALFF calculation) in the frontal, temporal, and occipital regions in the slow-5 band which were higher than those in the slow-4 band in the healthy subjects, while the slow-4 ALFF value was higher throughout the thalamus and basal ganglia. Combined with these results, it is indicated that the lower frequency band (slow-5, 0.010–0.027 Hz), which exhibits higher power, localizes mainly in the frontal, parietal, and occipital cortex. The higher frequency band (slow-4, 0.027–0.073 Hz), which has lower power, localizes more in subcortical structures [27, 64]. Our results added more clues to support this phenomenon.

According to the results shown by Figures 1 and 2, the results could be affected by both the disease (pulsatile tinnitus) and the ALFF analysis in different frequency bands. In order to make it clear if the ALFF changes are affected by the fact of disease (results of Figure 1) and/or the effect of different frequency bands (Figure 2), we performed a* post hoc* test to analyze the frequency-dependent changes in ALFF (Figure 3). Our results suggested that, for patients with pulsatile tinnitus, the abnormalities of brain activity were related to specific frequency bands (Figure 3). ALFF abnormalities in the aMPFC/ACC, PCu, right IPL, and some regions of occipital and parietal cortices and cerebellum exhibited group differences mainly in the slow-5 band results. It is indicated that the ALFF change in patients with PT was also frequency-dependent (slow-5/slow-4). This result suggested that slow-5 might be more sensitive in detecting PT-related neural changes. It was suggested that slow-4 is more prominent in the subcortical regions and slow-5 band ALFF is more prominent in cortical regions [27, 64–66]. ALFF alternations mainly occurred in the cortical regions according to previous studies [23, 24]. This might be the reason why slow-5 is more sensitive to the patients with this condition. Also, these results suggested that it is necessary to consider the effect of frequencies upon analyzing the rs-fMRI data of patients with pulsatile tinnitus. Frequency-dependent changes in ALFF were also detected in those patients with amnestic mild cognitive impairment [29], Alzheimer's disease [30], Parkinson's disease [31], schizophrenia [32], and so forth in several previous studies. These kinds of diseases were all closely related to abnormal neural activity. For PT patients, it was structural (focal defect of mastoid bone shell in the region of the transverse-sigmoid junction) rather than functional abnormalities that account for the etiologies. But we could also detect frequency-dependent changes in PT patients. This means that the pulsatile tinnitus could also lead to functional abnormalities in the brain, which should be paid special attention in the clinic.

Also, upon studying the functional connectivity network of the brain, seed/ROI-based functional connectivity analysis is one of the popular methods. But this method is highly dependent upon prior study and hypothesis to choose the seed/ROI. But there are limited ways to determine the number and location of the seed/ROI, making it vulnerable to bias. But the altered slow-4 and slow-5 ALFF activity might be indicators of the chosen seed/ROI. It was reported that brain activity in lower frequency bands may allow for interactions of neuronal networks [26]. In one of our recent published articles [25], increased short-ranged/long-ranged functional connectivity density (FCD: higher FCD value means increased functional connectivity between different brain areas) in PCu, right IPL, and so forth was found in the PT patients compared with normal controls. Similar alternations in these brain areas (PCu, right IPL, etc.) were also found in slow-4 and slow-5 bands in this study. The highly overlapping results between the two studies indicated that the frequency-dependent ALFF study results, especially the slow-5 results, set bases for further brain network studies.

## 5. Conclusion

In this study, we demonstrated widespread ALFF changes of neural activity in PT patients. The brain function abnormalities in PT patients exhibited different spatial patterns in different frequency bands. The slow-5 band might be more prominent in detecting PT-related neural changes. Additionally, the changed ALFF in PCu in both slow-4 and slow-5 bands were significantly correlated with THI score in patients with PT. The frequency-dependent ALFF study could help to set bases for functional connectivity studies. Taken together, our results indicate that a properly chosen frequency band can be more helpful in exploring PT-related neural changes. These findings may be helpful in uncovering abnormal neural activity in patients with PT at specific frequency bands.

## Figures and Tables

**Table 1:** Characteristics of the participants.

	PT (*n* = 40)	HC (*n* = 40)	*P* value
Age (year)	23–58 (36.0 ± 12.7)	23–58 (38.3 ± 11.5)	0.574^b^
Gender (male/female)	3/37	3/37	1.000^a^
Education (years)	4–16 (11.8 ± 3.7)	4–19 (12.7 ± 4.1)	0.168^b^
Handedness	40 right-handed	40 right-handed	1.000^b^
PT duration (months)	6–78 (30.9 ± 17.6)		
THI score	18–97 (53.6 ± 25.1)		

Data are presented as the range of min–max (mean ± SD). PT, pulsatile tinnitus; HC, healthy controls.

^a^Fisher's exact test.

^b^Two-sample *t*-tests.

## Additional Points

There are some limitations in this study. Firstly, we only enrolled patients with right-sided PT. PT patients only account for 4% among all of the persistent tinnitus patients [67]. So, actually, it is a relatively uncommon disease. Also, the etiology of “focal bone defect in the region of the sigmoid sinus” was only one of several etiologies of PT. There were many other kinds of etiologies that could induce PT. As a result, it is not easy to find enough PT patients with homogeneous symptoms (unilateral sided) and similar etiologies. For this reason, we only enrolled forty right-sided PT patients in our study. Secondly, the relationships between slow-5 and slow-4 bands were still not quite clear. The distinction between these two frequency bands has been described in many studies. There were studies in healthy subjects [27, 28] that provided us with more information about the spectral effects of fMRI. However, the exact clinical relevance of different frequency bands in different diseases was not fully described [29–31]. In this study, we carefully defined the inclusion and exclusion criteria and enrolled limited numbers of patients with pulsatile tinnitus. However, there were few patients with a THI score around 60 (from 52 to 70). It means that pulsatile tinnitus patients with moderate-severe handicap in this study were not a lot, which is a limitation. We have included this limitation in the last section of our paper. We will also pay more attention to include patients with different scale of severity in later studies. In a similar way, our research provided the different results in slow-5 and slow-4 bands in the PT patients. Analyzing BOLD signals at various spectra may provide additional information about the neural activity of the PT patients. Studies are still needed to understand the underlying physiological mechanisms of changed ALFF in each frequency band.
